# Stress as a Potential Regulatory Factor in the Outcome of Pharmacotherapy

**DOI:** 10.3389/fnins.2022.737716

**Published:** 2022-03-23

**Authors:** Maria Konstandi, Elizabeth O. Johnson, Matti A. Lang

**Affiliations:** ^1^Department of Pharmacology, Faculty of Medicine, University of Ioannina, Ioannina, Greece; ^2^Department of Anatomy, School of Medicine, European University Cyprus, Nicosia, Cyprus; ^3^Neomind Tech, Espoo, Finland

**Keywords:** stress, drug metabolism, cytochrome, CYP3A4, CYP2D6, pharmacotherapy

## Introduction

Accumulating clinical evidence suggests significant inter-individual variations in the efficacy of standard treatment protocols followed for several diseases, such as diabetes, hypertension, depression, cancer and epilepsy, as well as variations in drug-related side effects and toxicity (Zhou et al., 2009; Konstandi et al., 2014; Thummel and Lin, 2014). The problem often becomes more pronounced when multi-drug therapeutic schemes are followed (Konstandi, 2013; Konstandi et al., 2014; Thummel and Lin, 2014; Roughead, 2015). The reasons of this diversity appear to be related to the multi-factorial regulation of the machinery controlling the fate and biological activity of drugs in the body. Cell-signaling, metabolic and transport systems, which are encoded by their respective genes, participate in this machinery and in turn, are regulated by various factors including age, gender, race, lipidemic and endocrinological state (Ingelman-Sundberg, 2004a; Pelkonen et al., 2008; Waxman and Holloway, 2009). The functional integrity of the respiratory, immune, cardiovascular, gastrointestinal, endocrinological and central nervous systems also hold crucial modulatory roles in the machinery controlling drug activity (Ingelman-Sundberg, 2004b; Konstandi, 2013; Konstandi et al., 2014). External modifying factors such as stress stimuli, diet, environmental chemicals, toxicants and drugs, as well as infectious diseases can also modify the outcome and toxicity of pharmacotherapy by influencing the pharmacokinetic and pharmacodynamic profile of drugs (Zhou et al., 2009; Konstandi et al., 2014; Thummel and Lin, 2014; Roughead, 2015). This is attributed to the fact that they can affect the absorption, distribution, metabolism, elimination and activity of drugs (Zhou et al., 2009; Konstandi, 2013; Konstandi et al., 2013, 2014, 2020). In this context, stress plays a central role in the multi-factorial regulation of drugs in the body and in determining a drug's pharmacokinetic profile, as it regulates various enzymes that catalyze the metabolism of the majority of prescribed drugs (Konstandi et al., 2000, 2004, 2005, 2008, 2014; Daskalopoulos et al., 2012; Konstandi, 2013).

## Drug Metabolism

When a drug enters the body, it is recognized as a potential threat to homeostasis and the detoxifying mechanisms are activated (Handschin and Meyer, 2003; Konstandi et al., 2014), aimed at its metabolic conversion to usually inactive, water soluble metabolites, which can be readily excreted via urine or bile. The liver serves as the major site of drug metabolism, where enzymatic reactions catalyze the metabolic biotransformation of a drug typically in two phases: In Phase I, drugs are metabolized through various oxidation reactions to metabolites with increased water solubility. In Phase II, these metabolic products are conjugated with endogenous molecules, such as glucuronic acid, glutathione or sulfate groups, to form complexes with high water solubility (Gonzalez, 2005). The main families of enzymes that are involved in the metabolism of drugs during Phase I include cytochrome P450s (CYPs), flavin-containing monoxygenases (FMO) and epoxide hydrolases (EH). Enzymes of Phase II include glutathione S-transferases (GST), UDP-glucuronosyltransferases (UGT), *N*-acetyltransferases (NAT) and sulfotransferases (SULT) (Gonzalez, 2005). Depending on the structure of the drug, one or more of these enzymes catalyze its metabolism leading to modification of the drug's pharmacokinetic, pharmacodynamic and potentially toxicity profiles.

In several cases these enzymatic reactions can result in the formation of biologically active or toxic metabolites that can induce oxidative stress, cell death, carcinogenicity, teratogenesis or other toxic manifestations (Gonzalez and Gelboin, 1994; Guengerich, 2003; Ingelman-Sundberg, 2004a; Cribb et al., 2005; Gonzalez, 2005; Gonzalez and Yu, 2006).

Utilizing the metabolic activation, numerous pro-drugs have been developed over the past few decades, aiming at increased levels of biologically active molecules in the target tissues and less generalized toxic manifestations. Accordingly, pro-drugs are converted into pharmacologically active forms through metabolic activation that is mainly catalyzed by cytochromes (Chen et al., 2004; Gonzalez, 2005). This category of clinically important drugs include levodopa, talampicillin, cyclophosphamide, ftorafur, diazepam, prednisone, protonsil and enalapril, which are converted to dopamine, ampicillin, phosphoramide mustard, fluorouracil, oxazepam, prednisolone, sulfanilamide and enalaprilat, respectively (Sjovall et al., 1981; Chen et al., 2004; Rooseboom et al., 2004; Sozio et al., 2012; Konstandi, 2013).

### CYP-Dependent Drug Metabolism

Cytochrome (CYP) P450s are heme-containing proteins that are widely considered as the most important drug-metabolizing enzymes in humans and other animal species. They are able to collectively recognize and metabolize most structures, due to their broad and overlapping substrate specificities and are expressed virtually in all tissues, with generally highest concentrations and capacity in the liver for the main CYP isozymes. The main CYP isozymes catalyzing the metabolism of the majority of drugs presently in the market and other xenobiotics are arranged into three gene families (CYP1, CYP2, and CYP3) based on their amino acid sequence homology (Nebert, 2000; Nebert and Russell, 2002; Gonzalez, 2005). The most important human CYP isoforms are CYP1A1/2, CYP2A6, CYP2C8/9/19, CYP2D6, CYP2E1, and CYP3A4 (Wormhoudt et al., 1999; Lin et al., 2001; Nebert and Russell, 2002) that catalyze diverse oxidation reactions, including hydroxylations, heteroatom oxidations, heteroatom dealkylations, epoxidations, oxidative group transfer, cleavage of esters, and dehydrogenations (Hollenberg, 1992; Guengerich, 2008). They are also involved in the biosynthesis or catabolism of steroid hormones, neurotransmitters, bile acids, fat-soluble vitamins, fatty acids, and eicosanoids (Spatzenegger and Jaeger, 1995; Guengerich, 2003).

Inter-individual and inter-ethnic variability in drug response and adverse reactions, has been attributed in part, to the polymorphism of CYP *genes*, including CYP1A1, CYP2A6, CYP2A13, CYP2C8, CYP2D6, CYP3A4, and CYP3A5 (Gonzalez, 2005), and to variations in the distribution of the common allelic variants of CYP *genes* among different ethnic populations (Ingelman-Sundberg et al., 2007).

## Stress Impact on CYP-Dependent Drug Metabolism

The majority of the CYP *genes* are inducible and regulated by several external and internal factors, which, as a result, may also influence the fate and effects of drugs through modified enzymatic activity. Some of the emerging factors with increasing clinical significance, as demonstrated by various research groups, are psychophysiological stress and stress-related disorders, which appear to have a major impact on the expression and activity of several CYPs that catalyze the metabolism of widely prescribed drugs (Daskalopoulos et al., 2012; Konstandi, 2013; Konstandi et al., 2014). Our studies indicated that stress can affect constitutive and induced expression levels of CYP isoforms in ways that may critically modify the pharmacokinetic profile of drug-substrates (Konstandi, 2013; Konstandi et al., 2014) (https://www.fda.gov/drugs/drug-interactions-labeling/drug-development-and-drug-interactions-table-substrates-inhibitors-and-inducers). In particular, preclinical studies employing either early in life maternal deprivation stress, a neurodevelopmental model of stress, which is associated with various psychopathological states during adulthood (Rentesi et al., 2010, 2013) or repeated restraint stress, modified the hepatic drug-metabolizing profile of the animals in a stress-specific manner (Daskalopoulos et al., 2012). The stress-mediated regulation of CYP *genes* is a complex process involving several mechanisms, including transcriptional regulation through ligand-activated nuclear receptors, such as CAR, PXR and AhR (Nebert and Gonzalez, 1987; Gonzalez, 2005; Daskalopoulos et al., 2012; Konstandi, 2013; Konstandi et al., 2014). It also appears that stress activates major hepatic signal transduction pathways involved in CYP regulation, whereas long-term disturbances of these pathways can promote the accumulation of free radicals and other toxic metabolites in the body with potentially detrimental effects on health (Gonzalez, 2005; Konstandi et al., 2014).

The majority of studies focus on the impact of stress on the CYP-dependent drug metabolism during Phase I. It should be noted though, that stress could affect drug metabolism during Phase II, as it markedly reduces the glutathione content in tissues, when the body is exposed simultaneously to stress and in various toxic factors (Konstandi et al., 1998). This is a condition favoring the development of toxic manifestations, which usually lead to increased morbidity (Konstandi et al., 2014).

**Figure 1:**
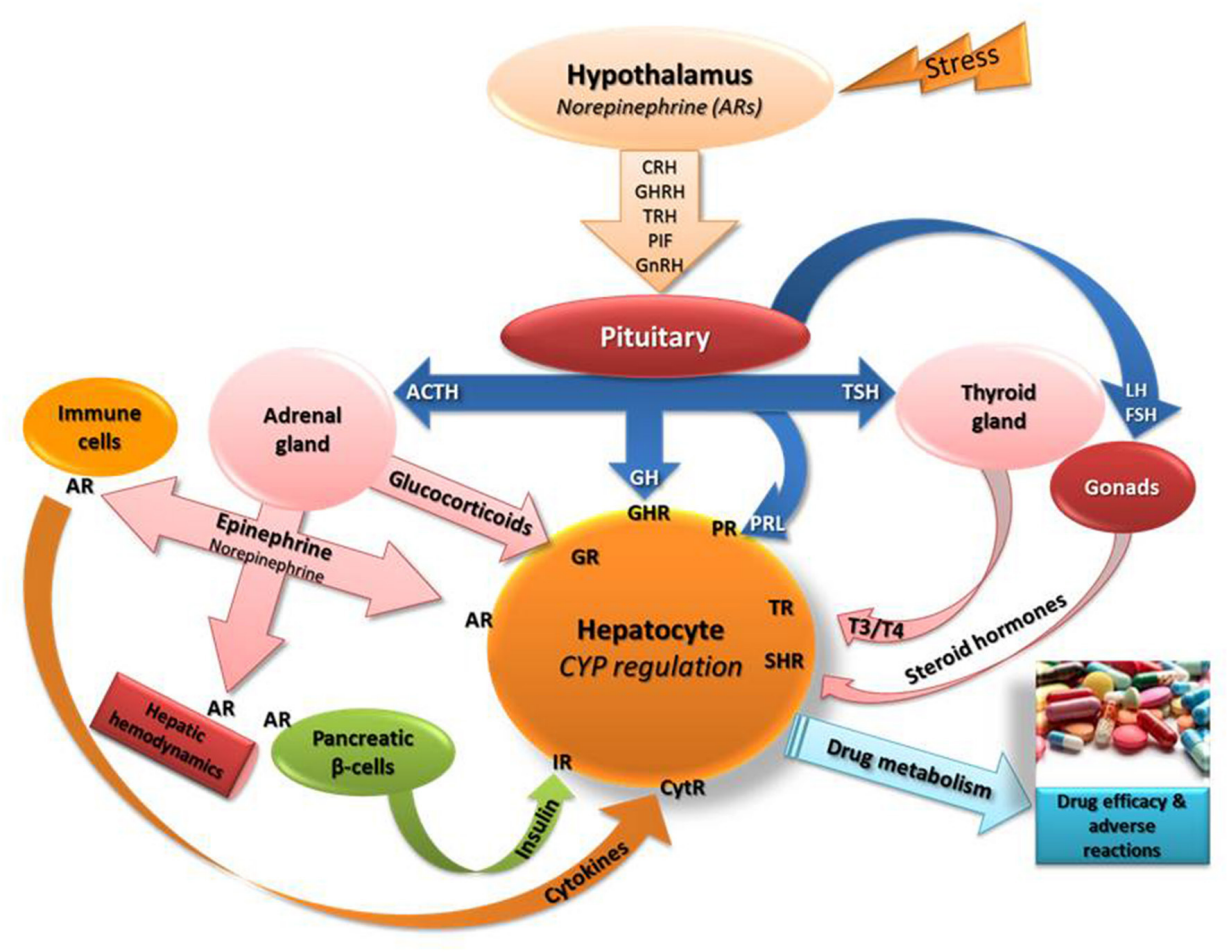
Schematic of the stress-induced neuroendocrine responses that presumably affect the regulation of the main drug-metabolizing cytochromes (CYP). Epinephrine and norepinephrine bind to adrenergic receptors (AR) in the periphery including hepatocytes, immune cells (stimulate release of cytokines), hepatic blood vessels (modify liver hemodynamics) and on pancreatic beta-cells (control insulin release positively via β2-ARs or negatively via α2-AR). Stress stimulates the secretion of norepinephrine in the hypothalamus that controls the release of several releasing factors regulating the secretion of hormones from the pituitary (Konstandi, 2013; Konstandi et al., 2014). The left side of the scheme indicates the effect of hypothalamic-pituitary-adrenal axis stimulation by stress on hepatic CYP regulation. CRH, corticotropin releasing hormone; GHRH, growth hormone releasing hormone; TRH, thyrotropin releasing hormone; PIF, prolactin inhibiting factor; GnRH, gonadotrophin releasing hormone; TSH, thyrotropin stimulating hormone; GH, growth hormone; T3, T4, thyroid hormones; ACTH, adrenocorticotropin hormone; PRL, prolactin; IR, insulin receptor; CytR, cytokine receptor; GHR, growth hormone receptor; TR, thyroid hormone receptor; GR, glucocorticoid receptor; SHR, steroid hormone receptor.

It is well documented that exposure to stress triggers various biological events in the body including primarily, activation of hypothalamo-pituitary-adrenal (HPA) axis followed by release of glucocorticoids and epinephrine from adrenal glands (Chrousos and Gold, 1992; Johnson et al., 1992; Chrousos, 2009; Chrousos and Kino, 2009; Figure 1). In the stress-induced cascade of events, oxidative stress, increased release of cytokines/NF-k and modifications in the secretion profiles of hormones, such as growth hormone, thyroid hormones and insulin (Dvorak and Pavek, 2010) hold critical roles in CYP regulation (Waxman et al., 1991; Woodcroft et al., 2002; Waxman and Holloway, 2009; Konstandi, 2013; Konstandi et al., 2014; Figure 1).

Although several studies clearly indicated that stress disrupts normal hepatic drug metabolism (Konstandi et al., 1998, 2000, 2004, 2005, 2014; Daskalopoulos et al., 2012; Konstandi, 2013) it is important to note that stress functioning as a modifying factor of drug metabolism is unique, with properties disparate to those of drugs, which usually have dose- and time-dependent specificities (Daskalopoulos et al., 2012; Konstandi, 2013; Konstandi et al., 2014). Usually, stress up-regulates the constitutive expression of most CYP enzymes, with the exception of *CYP2E1* and *CYP2B*, which are down-regulated. The stress-induced repression of these isozymes could decrease the metabolism of their drugs-substrates, thus resulting in elevated plasma levels of these drugs and consequently, increased possibility of toxic manifestations (Lang et al., 2001; Arinc et al., 2005; Gonzalez, 2005). On the other hand, it should be noted that psychophysiological stress up-regulates several CYP isozymes belonging to CYP1A, CYP2A, CYP2C, CYP2D, and CYP3A subfamilies that metabolize over 70% of the drugs in the market (Guengerich, 2008; Rendic and Guengerich, 2010; Konstandi, 2013; Konstandi et al., 2014). This induction could result in increased metabolism of their drugs-substrates, and consequently, in reduction of their efficacy. Of particular significance is the fact that stress upregulates CYP2D, which alternatively catalyzes the synthesis of neurosteroids and neurotransmitters, such as dopamine and serotonin, in the brain (Anna Haduch et al., 2013) and the hepatic and brain metabolism of the majority of antidepressant, antipsychotic, antiepileptic and anxiolytic drugs (Niwa et al., 2008; Rendic and Guengerich, 2010; Wang et al., 2014). *CYP2D* expression is also modified by these drugs and their effects are brain structure-dependent (Anna Haduch et al., 2013). The role of CYP2D in the pathophysiology of neurodegenerative disorders, such as Parkinson's disease, is currently under investigation (Tsuneoka et al., 1998; Mann et al., 2012; Ur Rasheed et al., 2017). From a clinical perspective, the impact of stress on CYP-catalyzed pro-drug metabolism is also very important. The stress-induced up-regulation of CYPs will result in increased pro-drug activation, whereas the *CYP* repression will result in reduced pro-drug activation, with respective consequences in drug efficacy (Konstandi, 2013; Konstandi et al., 2014).

The assessment of the effect of stress on drug metabolism, should not overlook the fact that chronic uncontrolled stress is considered as a causative factor in the pathogenesis of several disease states including cancer, depression, inflammatory diseases and those of metabolic syndrome, such as diabetes mellitus, obesity and hypertension (Gold et al., 1988; McEwen, 2000; Kloet et al., 2005). Patients suffering from these diseases have modified hormonal, immune and nutritional profiles compared to normal population (Chrousos and Gold, 1992; Tsigos and Chrousos, 2002), condition that could decisively affect their hepatic drug-metabolizing capacity (Konstandi, 2013; Konstandi et al., 2014). However, it remains to be determined whether the disease-related alterations in drug metabolism can be attributed to the disease itself, or they are associated with deregulation of the stress response system, which usually underlies the pathophysiology of the afore-mentioned diseases.

It is well established that the major effectors of the stress response, glucocorticoids and epinephrine, play primary, and partly distinct roles in the stress-induced regulation of CYPs by employing distinctive signaling pathways. Accordingly, drugs with sympathomimetic properties, or those acting as adrenergic receptor-blockers, or modifying the glucocorticoid, growth hormone, thyroid and insulin status, may influence the CYP-catalyzed drug metabolism, and therefore, the pharmacokinetics and pharmacodynamics of co-administered drugs and xenobiotics (Konstandi, 2013; Konstandi et al., 2014). The available evidence suggests that clinically applied drug dosing-regimes should be designed by taking into account possible drug-stress, drug-glucocorticoid and drug-adrenergic receptor interactions, which are known to modify drug efficacy and toxicity. Moreover, in addition to the pharmacologic profile of a drug, clinicians may consider the stress profile of the patient when determining the optimal dosing regime to ensure the highest possible drug efficacy and the minimum adverse reactions (Konstandi, 2013; Konstandi et al., 2014).

## Discussion

Increasing evidence suggests that psychophysiological stress plays a critical role in modifying the pharmacological and toxicological potency of many clinically used drugs by affecting the activity of CYP isozymes that catalyze their metabolism. Stress can affect the CYP-catalyzed drug metabolism in an enzyme- and stress-specific manner thus modifying the pharmacokinetic and pharmacodynamic profile of a drug and subsequently, the outcomes of drug therapy and toxicity. It is well documented that AR-linked pathways and glucocorticoids play major and partly, distinct roles in the stress-mediated regulation of CYPs. Although the mechanistic data have been obtained largely from preclinical studies, they provide solid evidence for the potential consequences of psychophysiological stress on drug metabolism in humans. This hypothesis is based primarily on the high similarity of the stress system functioning between mammals. It is therefore suggested that when designing a therapeutic scheme, particularly when it is based on multiple drugs, or on drugs with small therapeutic windows or with significant adverse effects, the patient's stress profile should be considered. In order to optimize the therapeutic efficacy of the drugs included in the prescribed scheme and minimize their side effects, elimination of stress is a prerequisite. Furthermore, when the treatment regimen includes drugs that affect the AR-linked pathways or the stress-related hormonal signaling, the drug dosing algorithms potentially need to be adjusted accordingly. Finally, the studies indicating the multi-faced and multi-level interplay between drug and stress system response, underscore the need of pharmacogenetic testing in the implementation of personalized medicine (Rostami-Hodjegan and Tucker, 2007; Zanger and Schwab, 2013).

## Author Contributions

MK drafted the original manuscript. EJ and ML critically revised the manuscript. All authors agree for the content of the work and approved it for publication.

## Conflict of Interest

ML was employed by Neomind Tech. The remaining authors declare that the research was conducted in the absence of any commercial or financial relationships that could be construed as a potential conflict of interest.

## Publisher's Note

All claims expressed in this article are solely those of the authors and do not necessarily represent those of their affiliated organizations, or those of the publisher, the editors and the reviewers. Any product that may be evaluated in this article, or claim that may be made by its manufacturer, is not guaranteed or endorsed by the publisher.

## References

[B1] Anna Haduch A. Bromek E. Daniel W. A. (2013). Role of brain cytochrome P450 (CYP2D) in the metabolism of monoaminergic neurotransmitters. Pharmacol. Rep. 65, 1519–1528. 10.1016/S1734-1140(13)71513-5 24553000

[B2] Arinc E. Arslan S. Adali O. (2005). Differential effects of diabetes on CYP2E1 and CYP2B4 proteins and associated drug metabolizing enzyme activities in rabbit liver. Arch. Toxicol. 79, 427–433. 10.1007/s00204-005-0654-8 15906000

[B3] Chen C.-S. Lin J. T. Goss K. A. He Y.-A. Halpert J. R.. (2004). Activation of the anticancer prodrugs cyclophosphamide and ifosfamide: identification of cytochrome P450 2B enzymes and site-specific mutants with improved enzyme kinetics. Mol. Pharmacol. 65, 1278–1285. 10.1124/mol.65.5.1278 15102956

[B4] Chrousos G. P. (2009). Stress and disorders of the stress system. Nat. Rev. Endocrinol. 5, 374–381. 10.1038/nrendo.2009.106 19488073

[B5] Chrousos G. P. Gold P. W. (1992). The concepts of stress and stress system disorders. Overview of physical and behavioral homeostasis. JAMA 267, 1244–1252. 10.1001/jama.267.9.1244 1538563

[B6] Chrousos G. P. Kino T. (2009). Glucocorticoid signaling in the cell. Expanding clinical implications to complex human behavioral and somatic disorders. Ann. N. Y. Acad. Sci. 1179, 153–166. 10.1111/j.1749-6632.2009.04988.x 19906238 PMC2791367

[B7] Cribb A. E. Peyrou M. Muruganandan S. Schneider L. (2005). The endoplasmic reticulum in xenobiotic toxicity. Drug Metab. Rev. 37, 405–442. 10.1080/03602530500205135 16257829

[B8] Daskalopoulos E. P. Malliou F. Rentesi G. Marselos M. Lang M. A. Konstandi M. (2012). Stress is a critical player in CYP3A, CYP2C, and CYP2D regulation: role of adrenergic receptor signaling pathways. Am. J. Physiol. Endocrinol. Metab. 303, E40–54. 10.1152/ajpendo.00545.2011 22510709

[B9] Dvorak Z. Pavek P. (2010). Regulation of drug-metabolizing cytochrome P450 enzymes by glucocorticoids. Drug Metab. Rev. 42, 621–635. 10.3109/03602532.2010.484462 20482443

[B10] Gold P. W. Goodwin F. K. Chrousos G. P. (1988). Clinical and biochemical manifestations of depression. Relation to the neurobiology of stress (1). N. Engl. J. Med. 319, 348–353. 10.1056/NEJM198808113190606 3292920

[B11] Gonzalez F. J. (2005). Role of cytochromes P450 in chemical toxicity and oxidative stress: studies with CYP2E1. Mutat. Res. 569, 101–110. 10.1016/j.mrfmmm.2004.04.021 15603755

[B12] Gonzalez F. J. Gelboin H. V. (1994). Role of human cytochromes P450 in the metabolic activation of chemical carcinogens and toxins. Drug Metab. Rev. 26, 165–183. 10.3109/03602539409029789 8082563

[B13] Gonzalez F. J. Yu A.-M. (2006). Cytochrome P450 and xenobiotic receptor humanized mice. Annu. Rev. Pharmacol. Toxicol. 46, 41–64. 10.1146/annurev.pharmtox.45.120403.100007 16402898 PMC1440291

[B14] Guengerich F. P. (2003). Cytochromes P450, drugs, and diseases. Mol. Interv. 3, 194–204. 10.1124/mi.3.4.194 14993447

[B15] Guengerich F. P. (2008). Cytochrome p450 and chemical toxicology. Chem. Res. Toxicol. 21, 70–83. 10.1021/tx700079z 18052394

[B16] Handschin C. Meyer U. A. (2003). Induction of drug metabolism: the role of nuclear receptors. Pharmacol. Rev. 55, 649–673. 10.1124/pr.55.4.2 14657421

[B17] Hollenberg P. F. (1992). Mechanisms of cytochrome P450 and peroxidase-catalyzed xenobiotic metabolism. FASEB J. 6, 686–694. 10.1096/fasebj.6.2.1537457 1537457

[B18] Ingelman-Sundberg M. (2004a). Human drug metabolising cytochrome P450 enzymes: properties and polymorphisms. Naunyn Schmiedebergs. Arch. Pharmacol. 369, 89–104. 10.1007/s00210-003-0819-z 14574440

[B19] Ingelman-Sundberg M. (2004b). Pharmacogenetics of cytochrome P450 and its applications in drug therapy: the past, present and future. Trends Pharmacol. Sci. 25, 193–200. 10.1016/j.tips.2004.02.007 15063083

[B20] Ingelman-Sundberg M. Sim S. C. Gomez A. Rodriguez-Antona C. (2007). Influence of cytochrome P450 polymorphisms on drug therapies: pharmacogenetic, pharmacoepigenetic and clinical aspects. Pharmacol. Ther. 116, 496–526. 10.1016/j.pharmthera.2007.09.004 18001838

[B21] Johnson E. O. Kamilaris T. C. Chrousos G. P. Gold P. W. (1992). Mechanisms of stress: a dynamic overview of hormonal and behavioral homeostasis. Neurosci. Biobehav. Rev. 16, 115–130. 10.1016/S0149-7634(05)80175-7 1630726

[B22] Kloet E. R. d. Joels M. Joels E. R. M. Holsboer F. (2005). Stress and the brain: from adaptation to disease. Nat. Rev. Neurosci. 6, 463–475. 10.1038/nrn1683 15891777

[B23] Konstandi M. (2013). Psychophysiological stress: a significant parameter in drug pharmacokinetics. Expert Opin. Drug Metab. Toxicol. 9, 1317–1334. 10.1517/17425255.2013.816283 23834396

[B24] Konstandi M. Andriopoulou C. E. Cheng J. Gonzalez F. J. (2020). Sex steroid hormones differentially regulate CYP2D in female wild-type and CYP2D6-humanized mice. J. Endocrinol. 245, 301–314. 10.1530/JOE-19-0561 32171179 PMC7202972

[B25] Konstandi M. Cheng J. Gonzalez F. J. (2013). Sex steroid hormones regulate constitutive expression of Cyp2e1 in female mouse liver. Am. J. Physiol. Endocrinol. Metab. 304, E1118–1128. 10.1152/ajpendo.00585.2012 23548611 PMC3651618

[B26] Konstandi M. Johnson E. Lang M. A. Camus-Radon A. M. Marselos M. (2000). Stress modulates the enzymatic inducibility by benzo[alpha]pyrene in the rat liver. Pharmacol. Res. 42, 205–211. 10.1006/phrs.2000.0675 10945924

[B27] Konstandi M. Johnson E. O. Lang M. A. (2014). Consequences of psychophysiological stress on cytochrome P450-catalyzed drug metabolism. Neurosci. Biobehav. Rev. 45, 149–167. 10.1016/j.neubiorev.2014.05.011 24877684

[B28] Konstandi M. Johnson E. O. Marselos M. Kostakis D. Fotopoulos A. Lang M. A. (2004). Stress-mediated modulation of B(alpha)P-induced hepatic CYP1A1: role of catecholamines. Chem. Biol. Interact. 147, 65–77. 10.1016/j.cbi.2003.10.007 14726153

[B29] Konstandi M. Kostakis D. Harkitis P. Marselos M. Johnson E. O. Adamidis K.. (2005). Role of adrenoceptor-linked signaling pathways in the regulation of CYP1A1 gene expression. Biochem. Pharmacol. 69, 277–287. 10.1016/j.bcp.2004.09.024 15627480

[B30] Konstandi M. Lang M. A. Kostakis D. Johnson E. O. Marselos M. (2008). Predominant role of peripheral catecholamines in the stress-induced modulation of CYP1A2 inducibility by benzo(alpha)pyrene. Basic Clin. Pharmacol. Toxicol. 102, 35–44. 10.1111/j.1742-7843.2007.00154.x 17973897

[B31] Konstandi M. Marselos M. Radon-Camus A. M. Johnson E. Lang M. A. (1998). The role of stress in the regulation of drug metabolizing enzymes in mice. Eur. J. Drug Metab. Pharmacokinet. 23, 483–490. 10.1007/BF03189999 10323331

[B32] Lang T. Klein K. Fischer J. Nussler A. K. Neuhaus P. Hofmann U.. (2001). Extensive genetic polymorphism in the human CYP2B6 gene with impact on expression and function in human liver. Pharmacogenetics 11, 399–415. 10.1097/00008571-200107000-00004 11470993

[B33] Lin Y. Lu P. Tang C. Mei Q. Sandig G. Rodrigues A. D.. (2001). Substrate inhibition kinetics for cytochrome P450-catalyzed reactions. Drug Metab. Dispos. 29, 368–374. 11259318

[B34] Mann A. Miksys S. L. Gaedigk A. Kish S. J. Mash D. C. Tyndale R. F. (2012). The neuroprotective enzyme CYP2D6 increases in the brain with age and is lower in Parkinson's disease patients. Neurobiol. Aging 33, 2160–2171. 10.1016/j.neurobiolaging.2011.08.014 21958961

[B35] McEwen B. S. (2000). The neurobiology of stress: from serendipity to clinical relevance. Brain Res. 886, 172–189. 10.1016/S0006-8993(00)02950-4 11119695

[B36] Nebert D. W. (2000). Suggestions for the nomenclature of human alleles: relevance to ecogenetics, pharmacogenetics and molecular epidemiology. Pharmacogenetics 10, 279–290. 10.1097/00008571-200006000-00001 10862518

[B37] Nebert D. W. Gonzalez F. J. (1987). P450 genes: structure, evolution, and regulation. Annu. Rev. Biochem. 56, 945–993. 10.1146/annurev.bi.56.070187.004501 3304150

[B38] Nebert D. W. Russell D. W. (2002). Clinical importance of the cytochromes P450. Lancet 360, 1155–1162. 10.1016/S0140-6736(02)11203-7 12387968

[B39] Niwa T. Okada K. Hiroi T. Imaoka S. Narimatsu S. Funae Y. (2008). Effect of psychotropic drugs on the 21-hydroxylation of neurosteroids, progesterone and allopregnanolone, catalyzed by rat CYP2D4 and human CYP2D6 in the brain. Biol. Pharm. Bull. 31, 348–351. 10.1248/bpb.31.348 18310890

[B40] Pelkonen O. Turpeinen M. Hakkola J. Honkakoski P. Hukkanen J. Raunio H. (2008). Inhibition and induction of human cytochrome P450 enzymes: current status. Arch. Toxicol. 82, 667–715. 10.1007/s00204-008-0332-8 18618097

[B41] Rendic S. Guengerich F. P. (2010). Update information on drug metabolism systems−2009, part II: summary of information on the effects of diseases and environmental factors on human cytochrome P450 (CYP) enzymes and transporters. Curr. Drug Metab. 11, 4–84. 10.2174/138920010791110917 20302566 PMC4167379

[B42] Rentesi G. Antoniou K. Marselos M. Fotopoulos A. Alboycharali J. Konstandi M. (2010). Long-term consequences of early maternal deprivation in serotonergic activity and HPA function in adult rat. Neurosci. Lett. 480, 7–11. 10.1016/j.neulet.2010.04.054 20435091

[B43] Rentesi G. Antoniou K. Marselos M. Syrrou M. Papadopoulou-Daifoti Z. Konstandi M. (2013). Early maternal deprivation-induced modifications in the neurobiological, neurochemical and behavioral profile of adult rats. Behav. Brain Res. 244, 29–37. 10.1016/j.bbr.2013.01.040 23395600

[B44] Rooseboom M. Commandeur J. N. Vermeulen N. P. (2004). Enzyme-catalyzed activation of anticancer prodrugs. Pharmacol. Rev. 56, 53–102. 10.1124/pr.56.1.3 15001663

[B45] Rostami-Hodjegan A. Tucker G. T. (2007). Simulation and prediction of in vivo drug metabolism in human populations from in vitro data. Nat. Rev. Drug Discov. 6, 140–148. 10.1038/nrd2173 17268485

[B46] Roughead E. E. (2015). Multidrug interactions: the current clinical and pharmacovigilance challenge. J. Pharmacy Pract. Res. 45, 138–139. 10.1002/jppr.1101

[B47] Sjovall J. Magni L. Vinnars E. (1981). Bioavailability of bacampicillin and talampicillin, two oral prodrugs of ampicillin. Antimicrob. Agents Chemother. 20, 837–838. 10.1128/AAC.20.6.837 7325647 PMC181808

[B48] Sozio P. Cerasa L. S. Abbadessa A. Stefano D. I. A. (2012). Designing prodrugs for the treatment of Parkinson's disease. Expert Opin. Drug Discov. 7, 385–406. 10.1517/17460441.2012.677025 22494466

[B49] Spatzenegger M. Jaeger W. (1995). Clinical importance of hepatic cytochrome P450 in drug metabolism. Drug Metab. Rev. 27, 397–417. 10.3109/03602539508998329 8521748

[B50] Thummel K. E. Lin Y. S. (2014). Sources of interindividual variability. Methods Mol. Biol. 1113, 363–415. 10.1007/978-1-62703-758-7_17 24523121

[B51] Tsigos C. Chrousos G. P. (2002). Hypothalamic-pituitary-adrenal axis, neuroendocrine factors and stress. J. Psychosom. Res. 53, 865–871. 10.1016/S0022-3999(02)00429-4 12377295

[B52] Tsuneoka Y. Matsuo Y. Ichikawa Y. Watanabe Y. (1998). Genetic analysis of the CYP2D6 gene in patients with Parkinson's disease. Metab. Clin. Exp. 47, 94–96. 10.1016/S0026-0495(98)90199-8 9440484

[B53] Ur Rasheed M. S. Mishra A. K. Singh M. P. (2017). Cytochrome P450 2D6 and Parkinson's Disease: Polymorphism, Metabolic Role, Risk and Protection. Neurochem. Res. 42, 3353–3361. 10.1007/s11064-017-2384-8 28871472

[B54] Wang X. Dong L. i. J Yue G. (2014). The endogenous substrates of brain CYP2D. Eur. J. Pharmacol. 724, 211–218. 10.1016/j.ejphar.2013.12.025 24374199

[B55] Waxman D. J. Holloway M. G. (2009). Sex differences in the expression of hepatic drug metabolizing enzymes. Mol. Pharmacol. 76, 215–228. 10.1124/mol.109.056705 19483103 PMC2713118

[B56] Waxman D. J. Pampori N. A. Ram P. A. Agrawal A. K. Shapiro B. H. (1991). Interpulse interval in circulating growth hormone patterns regulates sexually dimorphic expression of hepatic cytochrome P450. Proc. Natl. Acad. Sci. U.S.A. 88, 6868–6872. 10.1073/pnas.88.15.6868 1862110 PMC52190

[B57] Woodcroft K. J. Hafner M. S. Novak R. F. (2002). Insulin signaling in the transcriptional and posttranscriptional regulation of CYP2E1 expression. Hepatology 35, 263–273. 10.1053/jhep.2002.30691 11826398

[B58] Wormhoudt L. W. Commandeur J. N. Vermeulen N. P. (1999). Genetic polymorphisms of human N-acetyltransferase, cytochrome P450, glutathione-S-transferase, and epoxide hydrolase enzymes: relevance to xenobiotic metabolism and toxicity. Crit. Rev. Toxicol. 29, 59–124. 10.1080/10408449991349186 10066160

[B59] Zanger U. M. Schwab M. (2013). Cytochrome P450 enzymes in drug metabolism: regulation of gene expression, enzyme activities, and impact of genetic variation. Pharmacol. Ther. 138, 103–141. 10.1016/j.pharmthera.2012.12.007 23333322

[B60] Zhou S. F. Liu J. P. Chowbay B. (2009). Polymorphism of human cytochrome P450 enzymes and its clinical impact. Drug Metab. Rev. 41, 89–295. 10.1080/03602530902843483 19514967

